# Bacterial contamination potential of personal protective equipment itself in dental aerosol-producing treatments

**DOI:** 10.1007/s10266-023-00848-3

**Published:** 2023-09-13

**Authors:** Madline Priska Gund, Jusef Naim, Stefan Rupf, Barbara Gärtner, Matthias Hannig

**Affiliations:** 1 Department of Operative Dentistry, Periodontology and Preventive Dentistry, Clinic of Operative Dentistry, Saarland University Hospital, Saarland University, Kirrberger Str. 100, Building 73, 66421 Homburg, Saar Germany; 2 Institute of Medical Microbiology and Hygiene, Department of Hospital Hygiene, Saarland University, Homburg, Germany; 3 Chair of Synoptic Dentistry, Homburg, Germany

**Keywords:** Aerosols, Personal protective equipment (PPE), Contamination, Infection control, CHX

## Abstract

Personal protective equipment (PPE) has long been a high priority in dental aerosol-producing treatments. Since COVID-19 pandemic, its importance has increased yet again. While importance of PPE in preventing transmission and thus possible infection of pathogens is well known, contamination potential of PPE after treatment itself is less investigated. This review aims to give an overview of the current literature and contamination potential (viral, blood, bacterial) of components of protective equipment itself. The literature search was performed using the Medline database; furthermore, a hand search was conducted. Last search took place on 23 November 2022. Two categories of hygiene-related keywords were formed (category A: mask, face shield, goggles, eyewear, personal protective equipment; category B: contamination, aerosol). Each keyword from one category was combined with all keywords from the other one. In addition, the keyword “dental” was always added. First, a title and abstract screening was performed. Afterward, a full-text analysis was followed for the included studies. A total of 648 search hits were found in the Medline database. 47 were included after title and abstract screening. 22 studies were excluded after full-text analysis, 25 studies were included. The hand search resulted in 4 studies that were included. Bacterial contamination of PPE after treatment has been adequately studied, contamination with blood less. Microorganisms mainly originate from the oral and cutaneous flora; however, a transmission of potential pathogens like Staphylococcus aureus or *Escherichia coli* was also described. Studies showing transmission pathways starting from PPE and its various components are lacking. No measures have yet been described that fully protect the protective equipment from contamination. There is growing awareness that PPE itself can be a source of pathogen transmission, and thus possible infection. Therefore, not only wearing of protective clothing, but also conscious handling of it is crucial for transmission and possible infection prevention. However, studies showing transmission pathways starting from PPE and its various components are lacking. Several studies have investigated what measures can be taken to protect the protective equipment itself. So far, none of the methods evaluated can prevent contamination of PPE.

**Supplementary Information:**

The online version contains supplementary material available at 10.1007/s10266-023-00848-3.

## Introduction

### Personal protective equipment (PPE) in dentistry

During dental treatments, bioaerosols are generated contaminating the dentist, assistant, patient, and environment. For this reason, various items of personal protective equipment (PPE) have been worn as standard in dentistry for a long time. The following have become established as minimal equipment: goggles protecting the eyes, gloves the hands, and surgical masks patient and dentist himself. The standard can be expanded by: protective clothing protecting the body, face shields protecting additional areas of the face not covered by masks, and operation caps protecting the hair.

In daily practice, PPE is often reduced to a minimum (surgical mask, gloves, and occasionally protective eyewear). Obviously, many years before COVID-19 pandemic, PPE was already given a high priority in context of aerosol-producing dental treatments. The aim is to prevent contamination, transmission and, in addition, possible infection with or by pathogens through patient contact or objects and materials used to protect the dentist, assistant, and patient.

Nevertheless, intensification of PPE has been recommended since COVID-19 pandemic (operation cap, protective clothing, FFP-2 mask under surgical mask, and face shield) [1–3].

Surgical masks (type II) and FFP-2 masks differ considerably. Depending on microorganisms and fit of surgical mask, protection against aerosol infections may be severely compromised. However, the surgical mask provides effective protection against droplets. Since this applies to emission of infectious droplets by the wearer, the surgical mask is considered primarily as an external protection. However, this does not mean that the mask is not providing adequate self-protection if it fits tightly. Therefore, the correct fit is crucial, as the vertically folded shape means there is a risk that unfiltered air can be inhaled. Surgical masks mainly protect the environment and surrounding people. If they fit tightly, they also offer a certain degree of self-protection, protecting the dentist more effectively against droplets than aerosols. The vertically folded shape can affect the tight fit [4]. If the tight fit is not given, the protective effect can be significantly reduced, as unfiltered ambient air can be inhaled [4, 5].

In contrast, the FFP-2 mask (“Filtering Face Piece” mask), also known as particle-filtering half masks, provides foreign and self-protection from particles, droplets, and aerosols, being superior to surgical masks [6]. They are originally known from the field of crafts as “dust protection masks” [4]. With an integrated exhalation valve, the FFP mask primarily serves as self-protection. Due to aqueous base of dental spray mist, FFP-2 masks are recommended instead of type II surgical masks for dental treatments during COVID-19 pandemics [1–3, 7]. FFP masks can be divided into three categories depending on the filtering performance of particles (> 0.3 μm): FFP-1 masks with a filtering performance of > 80%, FFP-2 masks of > 94%, and FFP-3 masks of > 99%.

### Cutaneous and oral microbiota

The human oral microbiome database estimates the presence of prokaryotes in the oral cavity to be around 700–800 different species [8, 9]. The total number of viable bacteria averages about 10^8^ bacteria per ml of saliva [10]. The oral flora of a healthy person is dominated by streptococci, other commonly detected genera include Haemophilus, Neisseria, Prevotella, Veillonella and Rothia [9, 11], furthermore *Staphylococcus aureus* [12]. Commensals live in numerous niches provided by the oral cavity, such as the tongue, hard palate, cheeks, gums, soft palate and supra- and subgingival tooth surfaces. In addition, esophagus, pharynx, Eustachian tube, trachea, middle ear and nasal passages with paranasal sinuses serve as adjacent habitats also influencing the oral microflora [13].

Like the oral cavity, the skin offers many different niches where microorganisms are exposed to different ecological stresses. Characteristics of the niche determine the prevalence of the resident flora. However, the majority of bacteria identified are corynebacteria, propionibacteria, and staphylococci. Staphylococci predominate on the face and propionibacteria on sebaceous glands [14, 15]. Staphylococci also include the opportunistic pathogen *S. aureus*. In the global population, about 20% of healthy people are permanent carriers, 60% of people are intermittent carriers, and the remaining 20% are not colonized by *S. aureus*. The preferred habitat is the region of the anterior nasal vestibule to the posterior nasopharynx [16]. *S. aureus* is a common cause of morbidity and mortality worldwide, being responsible for a variety of moderate to severe diseases such as skin infections, sepsis, and pneumonia. Treatment is difficult due to antibiotic resistance and lack of a vaccine [17].

### Formation of bioaerosols and its contamination potential

In dentistry, highest speed instruments are used causing temperatures above 42.5 °C, potentially causing irreversible damage to the pulp chamber. Above 52 °C, the pulp tissue can even become necrotic [18]. Therefore, the pulp–dentin complex must always be protected from thermal damage by cooling instruments with water [19]. This results in spray mist consisting of an inhomogeneous mixture of water, tiny solids, and air, visible to the naked eye [20].

Spray mist can be divided into droplets and aerosols. Since droplets are larger than 5 µm, they cannot travel long distances and are subject to the evaporation process reducing the particle size and thus creating secondary aerosols. Aerosols are smaller than 5 µm, remain in the air for several hours and can be detected far away from the source [21].

In their review, Innes et al. also suggested that aerosols and droplets can contaminate the dental workplace, the practitioner, and assistant themselves during a wide range of dental treatments—from dental prophylaxis to invasive oral surgery [22].

Moreover, Timmerman et al., Singh et al., and Pasquarella et al. compared contamination levels before and during treatment uniformly detecting out that microbial load was highest during treatment itself [23–25].

Cristina et al. were able to show that blood can be regularly detected in dental aerosols [26]. Aerosols containing microorganisms are called bioaerosols [27, 28]. The microorganisms mainly originate from patient-related sources such as biofilm, calculus, blood, saliva, and the nasopharynx [29, 30]. Therefore, high concentrations of streptococci [30], staphylococci [31], and propionibacteria, being part of the natural microflora of skin and oral cavity, are also detected during treatment [32–34]. However, there are other non-patient sources forming bioaerosols such as general air contamination [35] and contaminated water pipes of treatment units [36]. Contaminated water pipes can pose a serious risk to immune-compromised patients, but also to dental staff, as the microorganisms present can be very diverse and potentially pathogenic [36]. Furthermore, potentially pathogenic microorganisms can often be isolated [29].

In summary, microorganisms from the patient and other non-patient-related sources can enter the ambient air during dental treatments forming the bioaerosols [37]. In general, these microorganisms are considered as non-pathogenic. Nevertheless, life-threatening infections are possible in vulnerable individuals with dysbiosis of the microbiome or impaired immune response [38]. Exposure to aerosols and droplets, harboring a wide variety of pathogenic microorganisms, also creates an increased risk of infection for dental staff [39]. Therefore, adequate protective measures are sensible and necessary.

The purpose of this narrative review was to evaluate the contamination potential of the personal protective equipment after dental aerosol-producing treatments.

## Methods

The literature search was conducted using the Medline database; furthermore, a hand search was conducted. The last search took place on 23 November 2022. Two categories with keywords were formed. Each keyword from one category has been combined with all keywords from the other one. In addition, the keyword “dental” was always added. First, a title and abstract screening was performed. Afterward, a full-text analysis was performed for the included studies (Table 1).

**Table 1:** Combined keywords for literature search

Category A	Category B
Mask	Contamination
Face shield	Aerosol
Goggles	
Eyewear	
Personal protective equipment	

## Results

A total of 648 search hits were found in the Medline database. 47 were included after title and abstract screening. 22 studies were excluded after full-text analysis and 25 studies finally included. The hand search resulted in 4 studies that were included. Additional 4 reviews were considered. Finally, 25 studies and 4 reviews were included.

## Contamination potential of the personal protective equipment (PPE)

### Surgical mask

While there are clear guidelines for hand disinfection and use of gloves to comply with the hygiene protocol [40], little attention has been paid to contamination potential of other components of the personal protective equipment itself. In 2021, a German research group was able to show for the first time that a surgical mask worn during aerosol-producing dental treatment can itself be a source of bacterial contamination after treatment. Previously unused surgical masks served as controls. It was recommended to change the surgical mask after each patient and not to touch the used mask after treatment with hands or new gloves [33]. The contamination rate of surgical masks, on the other hand, has more been studied and compared in different ways in literature. The study group just mentioned also compared contamination of surgical masks with the forehead after aerosol-producing dental treatments finding out that forehead contamination with bacteria was significantly lower in comparison to the surgical mask used. This was attributed to natural protection mechanisms of the skin. In both studies, the detected bacteria belonged to the oral and cutaneous flora, also potentially pathogens like *S. aureus* could be found. The forehead before treatment and unused surgical masks served as controls [34]. Several studies with limited study quality and significance due to lack of controls have also addressed contamination rate of masks. They could all detect massive contamination on masks after aerosol-producing treatments in dentistry [41–43]. The spectrum of microorganisms in these studies was very similar. In addition to staphylococci and streptococci, *Pseudomonas* and *E. coli*, often detected in nosocomial bloodstream infections [44], were dominant. Furthermore, one of these studies was able to show that the outside of surgical masks was significantly more contaminated with bacteria and fungi than the inside [41].

Comparison of contamination of outside and inside of masks was also subject of several in vitro studies showing that the inner side of the mask is regularly contaminated during treatments [45–48]. In one of these studies, contamination on the outer surface and even on the inner surface of single-layered surgical masks could be detected by both the operator and assistant on the dummy head where cavity preparation was performed using filter papers to assess the spread of the spray [45]. Moreover, aerosol distribution could even be found on the inside of a KN95 mask for all users and all devices (air polishing with an airflow device or ultrasonic scaling) during simulated periodontal treatment on a mannequin with fluorescein salivation, even though an additional face shield was worn [47].

In addition to bacterial contamination by aerosols, contamination of masks with blood was also investigated. Aguilar-Duran et al. examined face masks and caps of oral surgeons and assistants for blood contamination during 101 aerosol-producing various surgical procedures. Almost half of the 202 samples from assistants and oral surgeons were contaminated though a face shield was worn. 18.8% were macroscopically contaminated with blood. Interestingly, in 40% of the cases, clinicians were unaware of blood contamination. Dentists were more contaminated than assistants. No controls were included. In this context, it was strongly recommended protecting the face during oral surgery procedures, especially when using rotating equipment [49].

Many dental procedures generate droplets and aerosols contaminated with blood and bacteria, possibly leading to disease transmission [50]. In context of the COVID-19 pandemic, a systematic review was published summarizing the effectiveness of respiratory protective equipment (RPE) including mask, face shield, respirator, and goggles as a barrier against aerosolized microbes finding out that they can curb the spread of infection among healthcare workers. However, effectiveness of filtration is limited by mask-fit factor, period of wear, wetness of masks, fabrication quality, airflow dynamics, and inhalant particle size [50].

### Protective eyewear

Goggles have been rarely studied so far. The few existing studies show that they regularly become contaminated during aerosol-producing dental treatments [47, 51]. One study in particular has dealt with this issue in detail investigating the quantitative saliva and blood contamination of protective eyewear during aerosol-producing dental treatments. Goggles were disinfected before treatment. Contamination with blood was detected in all samples, with the highest amount found after professional tooth cleaning. Macroscopically detectable contamination was detected on 60.4% of protective eyewear. Macroscopically clean protective eyewear contained up to 12% contamination with blood. It was recommended to wear protective goggles without fail and to disinfect them after each patient since disinfection was effective against blood and saliva contamination [51].

Especially since Corona pandemic, eye protection has become increasingly important during treatment. Unprotected eyes and unprotected mucous membranes increase the risk of contracting COVID-19, so eyes should be protected with goggles. In addition, proper handling of protective eyewear is important, as they are rarely changed and disinfected during routine wear. Regular disinfection of goggles to avoid cross-contamination is, therefore, advisable [28, 52].

### Face shield

Central areas of the face, especially the inner part of the eyes and the nasal area, are most contaminated with visible splashes during periodontal and prosthetic treatment. The zygoma is least contaminated, contamination of left and right side of face does not differ. Contaminated areas are significantly higher in periodontal treatments than in prosthetic ones. Protecting the face with a mask, goggles, and a face shield is, therefore, urgently recommended [53]. Further studies indicate the importance of face shields and their effect on reducing facial contamination [47, 54, 55].

### Other components of the personal protective equipment

Some clinical and in vitro studies have looked at contamination of PPE as a whole or little regarded parts.

Bacterial contamination on sleeves of scrub jackets is higher than on the chest, as is the case when using ultrasonic or air polishers. Aerosol contamination is produced even when examining the patient or during hand scaling [56]. Again, bacteria of the dermal and oral flora were detected (Staphylococcus, Micrococcus, Bacillus, Actinomyces, Corynebacterium).

Al-Eid et al. investigated 30 oral surgical procedures for removal of one or both impacted mandibular third molars for visually indiscernible blood contamination using luminol. Luminol is mainly used in forensics for detection of invisible traces of blood. The PPE was worn by the oral surgeon, the patient, and assistant. Disposable protective equipment was used. Blood contamination could be found in all PPE (face masks, eyewear, surgical gown, sterile gloves) used by clinicians except head caps and shoe covers. Furthermore, eyewear and chest drapes used by patients were contaminated. Gloves and face masks of the surgeon were contaminated in all treatment cases, protective eyewear in 26 cases, and surgical gown in 22 cases. For the assistant, gloves were contaminated in all treatments, mask and glasses in 24 cases, and surgical gown in 20 cases. They recommended disinfection of all clinical surfaces and mandatory PPE for doctors and patients during every procedure, as imperceptible blood contamination occurs even during minor surgical ones [57].

In addition to the clinical studies already described, in vitro studies have also been conducted to investigate the PPE. Watanabe et al. investigated contamination patterns by adenosine triphosphate (ATP) bioluminescence analysis before and after dental treatment (ultrasonic scaling, professional mechanical tooth cleaning) on masks fitted with a surgical face shield, chest, goggles, and doctor’s gowned right arm, as well as on patient’s goggles. ATP is a useful marker in living microorganisms such as bacteria, fungi, and protozoa. The ATP bioluminescence method has long been used in monitoring surface contamination in hospitals and the food industry. The research group indicated that the contamination on every surface tested increased significantly after dental treatment. They summarized that aerosols and splashes generated during dental treatment have the potential transmitting infections to dentist and patient [58].

Nóbrega et al. described, in their review, that microorganisms could be found on many parts of the PPE after aerosol-producing dental treatments such as scrub jacket, sleeves, masks, and face shields. Different types of microorganisms like bacteria (*Staphylococcus auricularis* and epidermidis), viruses, and fungi were found.

They recommend, therefore, usage of PPE and regular disinfection procedures [59]. Also, Chanpong et al. recommended to wear a full PPE during aerosol-producing treatments and to switch between patients. They examined the extent of splashing during aerosol-generating procedures (up to 120 s) with a melamine resin visible under UV light in dental staff using a dental mannequin. In addition, a patient cough was simulated. After treatments, splashes were detected on body, arms, face, and legs of the dentist and assistant. As expected, the cough produced more splashes than the short aerosol-producing treatments; furthermore, contamination was found on the crown of the head, shoes, and back of the dental personnel [60]. Kaufmann et al. demonstrated that practitioners clothing (gloves, shoe, shirt, cap) is always contaminated. Ultrasonic scaling resulted in less contamination than air polishing. Moreover, probe contamination decreased with increasing distance from patient's mouth for both devices. [47]. Chen et al. demonstrated that when teeth were cleaned with water containing red pigments, every single waterproof protective gown was contaminated [54].

Reske et al. examined all PPE (gloves, face mask, eye protection, disposable gown) during donning, with a fluorescent marker applied to palms and abdomen finding out that self-contamination regularly occurs when donning and doffing PPE. The highest frequency of protocol deviations was in hand hygiene and use of disposable gowns. Protocol deviations were significantly associated with fluorescence. Participants were scanned for baseline fluorescence. Areas with fluorescence were cleaned, if possible, otherwise it was not taken into account [61]. The study shows again that the PPE itself can be a source of contamination, therefore handling it needs to be trained.

## Are there measures to protect the personal protective equipment (PPE)?

In addition to contamination of PPE itself by aerosol-producing treatments, extent to which the PPE can be protected by further measures was also examined.

## Protection of masks and against aerosols by face shield and pre-procedural mouth rinse with CHX

Gund et al. investigated whether a pre-procedural mouth rinse with CHX, water or no rinse and an additional face shield can prevent contamination of surgical masks with bacteria. Contamination of masks could be reduced by CHX and a face shield, but not prevented. Five unused surgical masks worn for 120 min during simulated work on a dummy head served as negative controls [62]. The bacteria detected belonged to the oral and dermal flora. However, it was striking that the bacterial diversity was significantly lower in the group rinsing with CHX. Furthermore, *S. aureus* could only be detected in the group not rinsing and in the group rinsing with water. Available in vitro studies confirm these results showing that a face shield has no significant retention function against aerosols [63], contamination can occur even on the inside of masks during aerosol-producing dental treatments [45]. Here, alpha-hemolytic streptococci were found. A multidisciplinary review published in 2021 dealt profoundly with this question. Face shields can reduce aerosol inhalation rate by 96%, and for small aerosols, the reduction rate is lower at 68%. For a short time, face shields can reduce inhalation of large aerosol particles, while smaller ones remain in the air longer and therefore can overcome the face shield [64]. One study investigating face shield and mask contamination after teeth had been cleaned with water containing red pigments using an oversized face shield could show that the face shield was contaminated in all cases, but the mask not at all, neither outside nor inside. Oversizing the face shield could explain the different results [54].

Serban et al. also found that a pre-procedural CHX rinse reduces bacterial contamination on masks compared to the group rinsing with sterile water after scaling procedures. In this case, agar culture plates were attached to the mask. Interestingly, a higher DMFT or calculus index resulted in more contamination. The bacteria detected were not explained further, neither strain nor species [65].

## Protection of face shield by pre-procedural mouth rinse with CHX

Also, protection of face shields was investigated. A pre-procedural CHX rinse can reduce, but not prevent bacterial contamination of face shields during aerosol-producing dental treatments [66]. Again, oral and cutaneous flora could be observed.

## Are there any other measures to protect the protective equipment?

As a way to reduce SARS-CoV-2 transmission in dentistry and reduction of contamination of PPE (surgical gloves, aprons, face shield), a new protective device consisting of a rigid, translucent acrylic structure and a suction tube encompassing the patient’s neck, head, and chest adapted to the dental chair can be used and was investigated in an in vitro study via dye during simulated dental procedures. With the device, dye could only be found on surgical gloves and fists (apron) [55]. However, it should be noted that the selected protective device is unhandy for everyday use and also not patient compatible. With a suction device (perioral suction device, Oral BioFilter) for perioral aerosol deposition during dental hygiene treatment, contamination of face shields can be prevented as one study showed [67].

## Conclusion and outlook

Especially since COVID-19 pandemic, the great importance of PPE for safe dental treatment has again become apparent. Bacterial contamination of PPE after treatment has largely been adequately studied, whereas there are only few studies on contamination with blood. In vitro studies were also conducted. Microorganisms mainly originate from the oral and cutaneous flora; nevertheless, a transmission of pathogens cannot be ruled out. *S. aureus* could be found on surgical masks after dental aerosol-producing treatments, a potentially pathogenic and multi-resistant bacterium. Also, *Pseudomonas* and *Escherichia coli*, are both frequently detected in nosocomial bloodstream infections.

They can be life threatening in vulnerable patients such as elderly, chronically ill or immunosuppressed ones. It is important to note that healthy patients were intentionally treated in these studies to reduce the risk of infection. It is very likely that the real risk from potentially pathogens is significantly higher.

In this context, growing awareness that PPE itself can be a source of pathogen transmission and therefore possible infection has developed. It has already been demonstrated that masks can be a source of contamination after dental aerosol-producing treatments. Therefore, recommendation was made that it should be changed after each treatment and not touched with new gloves or hands. Furthermore, it should not be placed on surfaces. However, studies showing transmission pathways starting from PPE and its various components are lacking. Moreover, several studies have also investigated what measures can be taken to protect the PPE itself. So far, none of the methods evaluated (e.g., face shield, pre-procedural mouth rinse with CHX) can prevent, but only reduce contamination of PPE. It must be noted in this context that face shields have no retention function against aerosols. In principle, measures must be suitable for everyday practice at low cost. At this stage, there is no way to make any practical recommendations. Further research is required.

### Supplementary Information

Below is the link to the electronic supplementary material.

Supplementary file1 (DOCX 25 KB)

## Data Availability

Not applicable.
