# Supplementary material for: Bacterial contamination potential of personal protective equipment itself in dental aerosol-producing treatments
Source: Odontology. 2023 Sep 13;112(2):309–16. doi: 10.1007/s10266-023-00848-3 (PMC10925564; doi:10.1007/s10266-023-00848-3)
Supplement: Supplementary file 1 — Supplementary file1 (DOCX 25 KB) [file 10266_2023_848_MOESM1_ESM.docx]

Table 2: Studies included in the review with brief description. Reviews are not included in the table.

In-vivo studies:

| **Study** | Short description | Methods |
| --- | --- | --- |
| **Contamination of surgical mask during aerosol-producing dental treatments** – [33] | All surgical masks (n=50) and all gloves (n=50) used during dental treatment displayed bacterial contamination.  Used masks have a potential to be a source of bacterial contamination for new gloves. | Used masks and gloves after treatment were imprinted on agar plates. Moreover, before masks were tested, their exterior surface was brought into contact with a unused sterile glove. This glove was also imprinted on agar. Samples were incubated and analyzed for bacterial growth. |
| **Bacterial contamination of forehead skin and surgical mask in aerosol-producing dental treatment –** [34] | Bacteria were detected in 95 % of skin-swab of the forehead and 76 % of surgical mask samples during dental treatment (n=67). Contamination of mask samples correlated with oral bacteria. | Sterile nylon swabs were used before and after a treatment to compare the bacteria on the forehead. Additionally, used masks were brought into contact with agar plates after treatment.  Samples were incubated and analyzed. |
| **Is safeguard compromised? Surgical mouth mask harboring hazardous microorganisms in dental practice** – [41] | The exterior surface of surgical masks (n=240) used in dental practice had significantly higher bacterial and fungal contamination than on the interior surface. | Exterior and Interior surface of used masks after treatment (max. duration 30 min) were separated by sterile technique and put in a sterile container consisting of trypticase soy broth. Spread plate method were used. Samples were incubated and analyzed for bacterial and fungal growth. |
| **Risk of Contamination of Different Areas of Dentist's Face During Dental Practices** – [54] | Contamination of different areas of dentist's face shield (n= 144) was significantly different. Areas around nose and inner corner of eyes were most contaminated, zygoma least. No significant differences were observed between the left and right side of the face shield. | Each treatment lasted between 40 and 50 minutes, with an average duration of 44 minutes. At the end of the treatment, the face shield was removed from the dentist's face and dried at room temperature. The visible splashes and droplets that accumulated on the cellulose face shields during treatment were evaluated. |
| **CHX and a Face Shield Cannot Prevent Contamination of Surgical Masks** – [63] | All masks were contaminated during dental treatment (n=306).  Patients rinsing with chlorhexidine reduced significantly bacterial contamination on surgical masks in comparison with water-rinsing or no-rinsing. | Participants rinsed for 60 s with 0.1 % CHX or with water before treatment, and, for control, a non-rinsing group was included. Face shield covered the mask during treatment. After treatment masks were brought into contact with agar plates. Samples were incubated and analyzed. |
| **Blood spatter in oral surgery: Prevalence and risk factors** – [49] | Facial Masks (n=101) and caps (n=101) of oral surgeons and assistants were evaluated for blood spatter after different oral surgery procedures (n=101). In 46 % of samples blood particles were detected. | The Kastle-Meyer test was used to investigate the presence of blood on different PPE after oral surgical procedures |
| **Predictors of quantitative microbiological analysis of spatter and aerosolization during scaling** – [66] | Bacterial contamination while scaling procedures (n=80) were significantly lower when patients rinsed with chlorhexidine compared to water-rinse. A higher DMFT or calculus indes resulted in more contamination. | Air samples were taken before treatment on different petri plates (agar, blood agar, Sabouraud). For air sampling Microbiological air quality sampler-Oxoid was used. During scaling another sample was taken on blood agar culture plate attached to dentist’s mask. All plates were incubated 24 hours at 37 degree celsius. |
| **Perioral Aerosol Sequestration Suction Device Effectively Reduces Biological Cross-Contamination in Dental Procedures** – [68] | Contamination of dental operatory locations were observed: operator's face-shield, back of the surgical operator's-gloves, patient's safety-goggles, and instrumental table. Perioral suction device showed significant reduction of contamination, but contamination could be still detected. | Adenosine triphosphate bioluminescence test was used to assess contamination with microorganisms after treatment with and without extraoral suction device. Sampling was performed by chemically impregnated reagent cotton swabs to evaluate the relative light units. Immediately before and after treatment, the sample sites were cleaned with disposable gauze containing 70% alcohol. |
| **Evaluation of aerosol contamination during debonding procedures** – [67] | Contamination of face shields and the dental chair table were observed. Significant more contamination could be found during debonding procedures (n=42) compared to control treatments (n=36). Furthermore chlorhexidine rinse led to an insignificant bacterial reduction. | Blood agar plates were attached to the face shields and the dental chair table and were used for collecting the aerosol samples. Samples were incubated and analyzed for microorganism growth. |
| **Blood and saliva contamination on protective eyewear during dental treatmen**t – [52] | Standardized protective eyewear shields (n=53) worn during different aerosol-producing dental treatment modalities were analyzed for blood and saliva contamination. Macroscopically detectable contamination was found on 60.4% of protective eyewear surfaces. A contamination with blood was detected on all shields after dental treatment. Generally, the amount of detected saliva was very low. | Luminol solution was used to assess contamination with blood on the protective eyewear. A special forensic test paper was used to visualize saliva contamination. Measurement methods were standardized to evaluate the results. |
| **Detection of Visually Imperceptible Blood Contamination in the Oral Surgical Clinic using Forensic Luminol Blood Detection Agent** – [58] | Oral surgical procedures were performed under local anesthesia in a disinfected clinic, and personnel protective (PPE) equipment was used by the oral surgeon, dental assistant, and patients. After the treatment, clinical surfaces and PPE were evaluated for traces of visually imperceptible blood contamination. Blood contamination was detected in flooring below surgical field (86.67%), instrument tray, operating light, dental chair, and suction unit (100%). Except head caps and shoe covers, blood contamination was detected in all the PPE used by the clinical personnel, and the eyewear and chest drapes used by patients. | After oral surgical procedures, the operatory room and the PPE was examined for blood contamination using luminol. |
| **Bacterial contamination of scrub jackets during dental hygiene procedures** – [57] | Long-sleeved gowns (n=26) were worn during dental procedures to determine bacterial contamination on the dominant arm, non-dominant arm, and chest. The dominant arm was more contaminated than the non-dominant arm. Aerosol contamination occurs during dental hygiene procedures, including examination and hand scaling. The number of microorganisms is higher on the sleeves of the gowns than on the chest, and it is higher when ultrasonic or sonic scalers or air polishers are used. | Dental hygiene procedures were conducted. No antimicrobial prerinses were used.  Sterile milipore filters were taped with non-sterile masking tape to the washed scrub jackets at start of each patient’s appointment. Once the tape was removed, the filter was placed on a sterile blood agar plate (Tryptic Soy Agar with 5% sheep blood). A sterile cotton swab was used to detach the filters from the plate. Plates were incubated in an aerobic culture chamber at 37 degree celsius for 24 hours. No anaerobic culturing  was done.  Number of colonies, colony morphology, gram stain reaction and microscopic morphology was recorded. |
| **Microbial Aerosol Contamination of Dental Healthcare Workers’ Faces and Other Surfaces in Dental Practice** – [43] | Samples (n=191) were taken to assess contamination from masks (n=52), mobile trays (n=52), near spittoons (n=52), and lamps (n=35) during dental treatment. The highest contaminated surfaces were, in descending order, dental healthcare workers’ surgical masks, lamps, near spittoons, and mobile trays. | Sterile nitrocellulose filters were fixed in an aseptic manner different locations and PPE. The filters were fixed to surfaces with a thin film of removable glue. Filters were removed at the end of the treatment with sterile tweezers and placed in sterile Petri dishes for bacterial growth. Samples were incubated and analyzed. |
| **Prevalence of microbial colonization in the mouth mask used by the dental professionals** – [42] | Masks (n=100) were examined for bacterial contamination by dentists after 30 min of routine dental procedure. All masks examined were found to be contaminated. | After 30 min of treatment, sterile cotton swabs were dipped in sterile peptone water to swab the exterior surface of the surgical mask. The samples were incubated and analyzed. |
| **Evaluating spatter and aerosol contamination during dental procedures** – [45] | Bacterial contamination during dental treatment on patients were observed. Regular contamination could be observed on the chest, head and mask of the dental staff. | During the treatment of patients, agar plates were laid out in various locations of the room including attached to the PPE for the evaluation of bacterial contamination. Samples were incubated and analyzed. |

In-vitro studies:

| **Study** | Short description | Methods |
| --- | --- | --- |
| **Use of ATP bioluminescence to survey the spread of aerosol and splatter during dental treatments** – [59] | Contamination patterns on the mask, goggles, chest and gowned right arm of operators were investigated. Contamination on every surface tested increased significantly after dental treatment. | Ultrasonic scaling and professional mechanical tooth cleaning was performed on 10 students. Each procedure lasting 10 minutes.  Contaminating ATP levels were recorded. They were expressed in relative light units. |
| **Assessment of dental health care personnel protocol deviations and self-contamination during personal protective equipment donning and doffing** – [62] | Dental health care personnel (n=70) self-contamination was common with donning and doffing personal protective equipment. | Fluorescent markers were applied to the abdomen and palms of the subjects. PPE donning and doffing was observed, and contaminated areas were assessed. |
| **Dissemination of aerosol and splatter during ultrasonic scaling: a pilot study** – [48] | Maximum contamination was found on the right arm of the operator and left arm of the assistant during ultrasonic scaling. Contamination was also found on the head, chest and inner surface of the face mask of the operator and of the assistant. | In a mannequin, ultrasonic application was performed for 15 minutes. A fluorescent dye was added to the water system. Filter paper discs at different positions and on the PPE were used to evaluate the spread of contamination. |
| **Assessment of dental personal protective equipment (PPE) and the relationship between manual dexterity and dissemination of aerosol and splatter during the COVID-19 pandemic** – [55] | Dental personnel wore leg covers, shoe covers, medical masks, haircaps, full masks, waterproof barrier gowns, and gloves. With exception of shoe covers, haircaps, and medical surgical masks, contamination were found on both the dentist and assistant's personal protective equipment. | Food pigment was added to the water outlet pipe of the treatment unit to visually assess the spread and contamination of the PPE. |
| **Dissemination of Aerosol and Splatter in Clinical Environment during Cavity Preparation: An In Vitro Study** – [46] | Contamination of the operators and assistants chest, head, forearms, upper leg and the inside of the face mask could be detected. The contaminated body surface area of the operator was more than that of the assistant. | Cavity preparation was performed on a mannequin. A fluorescent dye was added to the water system. To assess the spread of contamination, filter paper discs were used at different positions and on the PPE. |
| **Protective device to reduce aerosol dispersion in dental clinics during the COVID‐19 pandemic** – [56] | A device consisting of a rigid, transparent acrylic structure containing the head, neck and chest of the mannequin was used to investigate contamination during dental procedures. Without the device, contamination was detected on surgical gloves, aprons, and face shields. Using the device, contamination was noted only on the surgical gloves and apron. The dentist's freedom of movement and visualization with the device were limited. | With and without the device, a treatment was simulated in the 11 o'clock position for 60s. A flurorescent dye was added to the water system of the dental unit. The spread of the dye was evaluated at different locations and on the PPE. |
| **Aerosol-Generating Procedures and Simulated Cough in Dental Anesthesia** – [61] | The extent of splatter on dental personnel that may occur with aerosol generating procedures (AGPs) and coughing in a dental anesthesia practice. After AGPs were performed, splatter was noted on the face, body, arms, and legs of the dentist and dental assistant. The simulated cough produced more extensive splatter than AGPs; additional contamination was observed on the shoes, the crown of the head, and the back of the dental personnel. | Dental treatments were simulated on a mannequin whose teeth were coated with a melanin resin that appears blue under UV light. A simulated cough was also created using a ventilator programmed to expel the melanin resin within the velocity and volume parameters of a natural cough. After treatment the spread of contamination with the melanin resin were evaluated. |
| **Quantitative measurements of aerosols from air-polishing and ultrasonic devices: (How) can we protect ourselves?** – [47] | A mannequin with was treated applying periodontal treatment options.  Practitioners clothing (gloves, shoe, shirt, cap) is always contaminated. Aerosol distribution could even be found on the inside of a KN95 mask, even though an additional face shield was worn.  Moreover, probe contamination decreased with increasing distance from patient's mouth. | A mannequin with simulated fluorescein salivation was treated with two different periodontal treatment options for 5 minutes each. The deposition of aerosol and splashes was quantitatively measured at predefined locations at different distances from the mannquin’s mouth, among others, on the PPE in triplicate using absorbent filter papers. |
| **Evaluating spatter and aerosol contamination during dental procedures** - [45] | Contamination was observed on the upper surfaces of the operator’s arms, on the chest and lower neck region, around the mannequin, face masks of the dental personnel, despite the use of a face shield. Contamination was also found on the inside of the single layered mask and was even detected in the nose of the operator and assistant. | In the treatment of a mannequin, a fluorescent dye was added to the water system to observe the dispersion of the aerosol and droplets. Filter paper discs were placed at different locations in the room and on the PPE. |
